# New Horizons in Peripheral Artery Disease

**DOI:** 10.1093/ageing/afae114

**Published:** 2024-06-15

**Authors:** John S M Houghton, Athanasios N Saratzis, Rob D Sayers, Victoria J Haunton

**Affiliations:** Department of Cardiovascular Sciences, University of Leicester, Leicester, UK; Leicester Vascular Institute, University Hospitals of Leicester NHS Trust, Leicester, UK; National Institute for Health Research Leicester Biomedical Research Centre—The Glenfield Hospital, Leicester, UK; Department of Cardiovascular Sciences, University of Leicester, Leicester, UK; Leicester Vascular Institute, University Hospitals of Leicester NHS Trust, Leicester, UK; National Institute for Health Research Leicester Biomedical Research Centre—The Glenfield Hospital, Leicester, UK; Department of Cardiovascular Sciences, University of Leicester, Leicester, UK; Leicester Vascular Institute, University Hospitals of Leicester NHS Trust, Leicester, UK; National Institute for Health Research Leicester Biomedical Research Centre—The Glenfield Hospital, Leicester, UK; Faculty of Health, University of Plymouth, Plymouth, UK

**Keywords:** peripheral artery disease, chronic limb-threatening ischaemia, vascular surgical procedures, frailty, cognitive dysfunction, older people

## Abstract

Peripheral artery disease (PAD) is the lower limb manifestation of systemic atherosclerotic disease. PAD may initially present with symptoms of intermittent claudication, whilst chronic limb-threatening ischaemia (CLTI), the end stage of PAD, presents with rest pain and/or tissue loss. PAD is an age-related condition present in over 10% of those aged ≥65 in high-income countries. Guidelines regarding definition, diagnosis and staging of PAD and CLTI have been updated to reflect the changing patterns and presentations of disease given the increasing prevalence of diabetes. Recent research has changed guidelines on optimal medical therapy, with low-dose anticoagulant plus aspirin recommended in some patients. Recently published randomised trials highlight where bypass-first or endovascular-first approaches may be optimal in infra-inguinal disease. New techniques in endovascular surgery have increased minimally invasive options for ever more complex disease. Increasing recognition has been given to the complexity of patients with CLTI where a high prevalence of both frailty and cognitive impairment are present and a significant burden of multi-morbidity and polypharmacy. Despite advances in minimally invasive revascularisation techniques and reduction in amputation incidence, survival remains poor for many with CLTI. Shared decision-making is essential, and conservative management is often appropriate for older patients. There is emerging evidence of the benefit of specialist geriatric team input in the perioperative management of older patients undergoing surgery for CLTI. Recent UK guidelines now recommend screening for frailty, cognitive impairment and delirium in older vascular surgery patients as well as recommending all vascular surgery services have support and input from specialist geriatrics teams.

## Key Points

- Peripheral artery disease (PAD) is more common in older people and is a major cause of morbidity and mortality.
- Recent research has changed guidelines on optimal medical therapy in PAD.
- Frailty, cognitive impairment, multi-morbidity and polypharmacy are common in PAD.
- Newer technologies are offering minimally invasive options for increasingly complex disease.
- Access to specialist geriatric teams is a key component of care for patients with chronic limb-threatening ischaemia.

## Introduction

Peripheral artery disease (PAD) is the manifestation of systemic atherosclerotic disease in the arteries of, and supplying, the lower limbs [1]. Similar to other cardiovascular diseases, PAD is an age-related condition with a prevalence over 10% among people aged ≥65 living in high-income countries [2]. Whilst the majority of individuals with PAD are asymptomatic, progression of the disease leads to symptoms of intermittent claudication (leg pain on walking) and ultimately pain at rest, ulceration and gangrene. PAD is a major cause of morbidity among older people and may lead to disability, limb loss and death.

In recent years, there have been a number of innovations in medical and surgical management that have changed treatment algorithms for PAD. Additionally, new evidence and updated guidelines have radically changed management pathways with the advent of rapid-access limb salvage clinics to expedite revascularisation for those with rest pain and/or tissue loss [3–5]. In the context of an ageing population and increasing prevalence of type-2 diabetes mellitus, patient cohorts of those undergoing intervention have changed drastically with many more older patients with PAD living with multi-morbidity, frailty and cognitive impairment admitted to vascular surgery wards [6–10].

## Diagnosis of PAD

Clinical diagnosis of PAD is based on history (e.g. intermittent claudication) and examination findings (e.g. absent pedal pulses) as well as haemodynamic tests that are required to confirm the diagnosis [1]. The standard haemodynamic test is the ankle-brachial pressure index (ABPI), which is the ratio of systolic blood pressure in the ankle to that in the upper arm.

A resting ABPI <0.90 is considered the threshold for diagnosing PAD [11]. As well as identifying PAD in individuals with symptoms, ABPI can be used to diagnose those with asymptomatic PAD [11–13].

In individuals with diabetes or chronic kidney disease, ankle pressures may be falsely elevated or incompressible due to calcification of the crural (below knee) arteries [11]. Measurement of absolute toe pressure is preferable in these patients [5].

In clinical practice, arterial imaging (e.g. duplex ultrasound, computed tomography angiography) is reserved for those in whom revascularisation is being considered.

## Chronic limb-threatening ischaemia

Chronic limb-threatening ischaemia (CLTI) is the end stage of PAD, where the viability of the affected limb is threatened due to the degree of ischaemia in the distal tissues [5]. Previously termed critical limb ischaemia or severe limb ischaemia, the modern definition and staging of CLTI were developed to better reflect the spectrum of disease encountered in contemporary clinical practice in the context of increasing prevalence of diabetes. CLTI is defined as at least 2 weeks of ischaemic foot pain at night or at rest and/or tissue loss (ischaemic ulceration or gangrene) attributable to confirmed PAD [5].

In CLTI, severe PAD leads to a critical reduction in perfusion pressure in the distal tissues in the leg that cannot be overcome by compensatory mechanisms, such as arterial collateralisation and peripheral vasodilation [14]. Chronic microvascular changes also occur leading to haemostasis and microthrombosis, further exacerbating tissue ischaemia [14]. This tissue ischaemia leads to necrosis, directly causing ulceration and gangrene, predominantly in the digits and forefoot.

**Figure 1:**
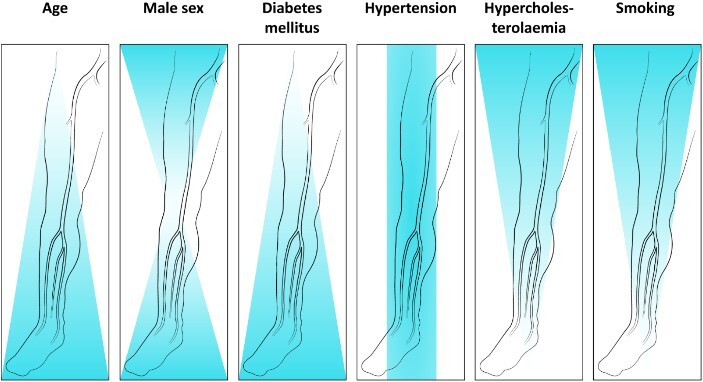
Association of risk factors with pattern of arterial disease (reproduced with permission from Diehm *et al.* 2006) [17].

Pathology and pattern of arterial disease can be different depending on the aetiology of PAD in an individual. Smoking and hypercholesterolaemia tend to be associated with atherosclerosis of the aortic bifurcation, iliac, common and superficial femoral arteries (above knee), whilst diabetes and older age are more frequently associated with disease affecting the crural arteries (Figure 1). Sensory, motor and autonomic neuropathy are also frequently present in those with long-standing diabetes, and diabetic foot disease itself is a spectrum of neuropathic and ischaemic causes. Biomechanical changes in the foot (causing foot deformity) and reduced sensation to pain and pressure lead to callous development and ultimately foot ulceration in load-bearing areas [5, 15]. Foot infection, due to relative immunosuppression from hyperglycaemia, is also both a cause and complication of diabetic foot ulceration [16].

Individuals with diabetes presenting with CLTI often have neuro-ischaemic foot ulceration. Therefore, optimisation of diabetes medications and offloading footwear to relieve pressure on areas of ulceration when mobilising and stringent wound care are essential elements of multi-disciplinary management of patients with diabetes-related CLTI in addition to revascularisation [5].

Staging of severity of CLTI is important in guiding management. The recently developed wound, ischaemia and foot infection (WIfI) classification reflects the spectrum of disease in CLTI encountered in modern practice: staging the disease based on extent of tissue loss, degree of ischaemia and presence and severity of concomitant bacterial infection [18]. Each component is scored 0–3 with the combined score given an overall clinical stage for both estimated risk of major lower limb amputation at 1 year and estimated likelihood of benefit of revascularisation (Table 1a and 1b).

**Table 1a:** Clinical stages of estimated risk of major amputation based on WIfI scores (reproduced with permission from Mills *et al.* 2014) [18]

Wound	Ischaemia—0				Ischaemia—1				Ischaemia—2				Ischaemia—3			
**0**	1	1	2	3	1	2	3	4	2	2	3	4	2	3	3	4
**1**	1	1	2	3	1	2	3	4	2	3	4	4	3	3	4	4
**2**	2	2	3	4	3	3	4	4	3	4	4	4	4	4	4	4
**3**	3	3	4	4	4	4	4	4	4	4	4	4	4	4	4	4
**Foot infection**	**0**	**1**	**2**	**3**	**0**	**1**	**2**	**3**	**0**	**1**	**2**	**3**	**0**	**1**	**2**	**3**

*Clinical stages (risk of amputation): 1 (very low risk); 2 (low risk); 3 (moderate risk);*

*4 (high risk).*

**Table 1b:** Clinical stages of estimated likelihood of benefit of revascularisation based on WIfI scores (reproduced with permission from Mills *et al.* 2014) [18]

Wound	Ischaemia—0				Ischaemia—1				Ischaemia—2				Ischaemia—3			
**0**	1	1	1	1	1	2	2	3	2	2	3	3	3	4	4	4
**1**	1	1	1	1	2	3	3	3	3	4	4	4	4	4	4	4
**2**	1	1	1	1	3	3	4	4	4	4	4	4	4	4	4	4
**3**	1	1	1	1	3	3	3	4	4	4	4	4	4	4	4	4
**Foot infection**	**0**	**1**	**2**	**3**	**0**	**1**	**2**	**3**	**0**	**1**	**2**	**3**	**0**	**1**	**2**	**3**

*Clinical stages (likelihood of benefit of revascularisation): 1 (very low likelihood of benefit);*

*2 (low likelihood of benefit); 3 (moderate likelihood of benefit); 4 (high likelihood of benefit).*

## Risk factors for PAD

As PAD is a manifestation of systematic atherosclerotic disease, it shares many of the same risk factors as cardiovascular disease [1]. More than 60% of individuals with PAD also have coronary artery or cerebrovascular disease, and people with PAD are at greater risk of myocardial infarction, cardiac death and stroke [19–21].

Age is the predominant risk factor for developing PAD. In all populations, the prevalence of PAD increases with age [2]. Smoking, diabetes mellitus, hypertension and hypercholesterolaemia are all risk factors for PAD [2, 19]. In those with diabetes, duration and worse glycaemic control are both associated with higher risk of developing PAD [22].

Current estimates are that over 235 million individuals are living with PAD worldwide [2]. Prevalence increases with age globally and across both sexes, with an estimated worldwide prevalence of PAD of 9% in those aged ≥65 rising to 25% among individuals aged ≥90 [2]. Prevalence of PAD is currently highest in high-income countries, although with rapidly improving life expectancy and increasing prevalence of smoking and diabetes, the prevalence of PAD among low- and middle-income countries is expected to rise [2, 7, 23].

The estimates of incidence and prevalence of CLTI are much less reliable than for PAD overall. Half of patients with CLTI will have had asymptomatic PAD prior to presenting with CLTI [24]. Furthermore, in those with symptomatic PAD, CLTI does not predictably progress from intermittent claudication with a wide range of estimates of between 12% and 29% progression from claudication to CLTI over 5 years [25]. The high risk of early mortality in those with CLTI and need for haemodynamic data to corroborate CLTI diagnosis (often lacking in population-based studies) mean the true incidence and prevalence of CLTI is likely underestimated [5]. It has been estimated that 11% of the PAD population have CLTI in the USA, with an estimated national population-wide incidence of 0.35% annually and prevalence of 1.35% among those aged ≥40 [26].

## PAD in older people

Older people with PAD have a high prevalence of geriatric syndromes such as frailty, cognitive impairment, multi-morbidity and polypharmacy [8, 9, 27]. In vascular surgery patients, including those with PAD, frailty is related to female sex, lower body mass index and a number of co-morbidities [28]. Both diagnosis of PAD and lower ABPI are associated with a higher prevalence and incidence of cognitive impairment [29]. As many as 50% of vascular surgery patients have cognitive impairment, including those with PAD [9].

Patients with PAD, particularly those with CLTI, often have disease-related lower limb dysfunction leading to disability [30]. Coupled with chronic inflammation from limb ischaemia, tissue loss and chronic infection (often present in CLTI), it is unsurprising that a higher proportion of those with more severe CLTI are living with frailty [8]. There is some evidence that, in a small proportion of patients, frailty trajectory may be slowed or reversed after successful revascularisation in those with CLTI and frailty [31].

PAD is a contributory cause of up to 20% of all leg ulcers in the older population and in people who are bed-bound or immobile, those with PAD are at higher risk of pressure damage to the heels [32–34].

## Medical management of PAD

The mainstay of medical management of PAD is risk factor modification through lifestyle change (e.g. smoking cessation), cardiovascular secondary preventative medical therapy with antithrombotic and statin therapy, lowering blood pressure in those with hypertension and optimising glycaemic control in those with diabetes [1].

### Antithrombotic therapy

There is good evidence that long-term antithrombotic therapy reduces cardiovascular events in those with symptomatic PAD [1]. Post-hoc analysis of the CAPRIE trial demonstrated that clopidogrel was marginally superior to aspirin in reducing major adverse cardiovascular events in patients with symptomatic PAD [35]. More recently, the COMPASS and VOYAGER PAD trials tested low-dose rivaroxaban (2.5 mg twice daily) plus aspirin versus aspirin plus placebo in patients with PAD. They demonstrated that combination therapy of rivaroxaban and aspirin compared to aspirin alone reduced both major cardiovascular events and major adverse limb events (including major amputation) in those with stable PAD (COMPASS) and major adverse limb events following successful revascularisation (VOYAGER) [36–38].

The lack of head-to-head comparison of rivaroxaban plus aspirin and clopidogrel in the management of patients with PAD makes decision-making regarding the optimal antithrombotic treatment difficult. Network meta-analysis of individuals with PAD from CAPRIE and COMPASS demonstrated no superiority of rivaroxaban and aspirin over clopidogrel [39]. Furthermore, both COMPASS and VOYAGER excluded patients with significant multi-morbidity and those considered at high risk of bleeding and, based on registry data, only 30% of patients hospitalised with symptomatic PAD would have been eligible for inclusion [40].

The recently published European guidelines recommend assessing all patients with symptomatic PAD for bleeding risk prior to selecting antithrombotic therapy [41]. Single agent clopidogrel is still recommended as first-line therapy in those with symptomatic PAD. However, in patients with PAD deemed to have both a lower bleeding risk and a higher risk of ischaemic events, low dose rivaroxaban plus aspirin should be considered [41]. These recommendations need to be applied carefully on an individualised basis, particularly in older patients. Furthermore, many patients with symptomatic PAD also have indications for long-term anticoagulation (e.g. those with atrial fibrillation) in whom the added benefit of antiplatelet therapy is unknown and is likely to significantly increase bleeding risk [40].

### Lipid-lowering therapy

There has been little change in recommendations for lipid-lowering therapy for those with PAD in recent years. Statins are recommended as secondary prevention for cardiovascular events in all those with PAD [1]. High-dose atorvastatin (80 mg once daily) is recommended for all patients with PAD by the UK National Institute for Health and Care Excellence (NICE) based on both network meta-analysis and cost-effectiveness analysis of randomised trials [42, 43]. Lower doses of atorvastatin should be considered in those with potential drug reactions, high risk of adverse events or based on patient preference [44].

### Exercise therapy

In addition to lifestyle modification and best medical therapy, exercise is effective at improving walking distance and quality of life in people with intermittent claudication. Guidelines recommend weekly supervised exercise therapy for 3 months based on meta-analysis of randomised trials [1, 43]. However, access to supervised exercise therapy is limited and not available for many patients [45]. Furthermore, uptake and adherence are poor even when available [45]. Guidelines now recognise that alternative exercise regimes and unsupervised/home-based exercise also have benefit [1].

Exercise therapy has no role in CLTI until after successful revascularisation, given CLTI-related disability and frequent need for urgent intervention for limb salvage.

### Medical management of CLTI

All CLTI patients should be considered for surgical or endovascular intervention for both revascularisation and wound management; however, medical management forms an important part of treatment.

Analgesia is required in all patients with rest pain, although no evidence exists of optimal regimens for effective pain relief [5]. Patients with concomitant infection will require antibiotics, with treatment guided by wound, fluid and tissue cultures as well as extent of infection [5]. Patients with CLTI-related foot infection, particularly those with diabetes, may have osteomyelitis, which requires a prolonged course of antibiotics if managed medically. As such imaging (plain radiograph +/− MR imaging of the foot) is needed in those with suspected deep infection.

## Surgical management of PAD

Individuals with lifestyle limiting claudication who have failed to significantly improve following best medical and exercise therapy may be considered for revascularisation; however, revascularisation is usually reserved for those with CLTI [1, 43]. This is particularly true in the older adult population in whom perioperative risk is higher and intervention is reserved for limb salvage alone.

In CLTI, individuals with diabetes who have predominantly neuropathic ulceration with minimal ischaemia (WIfI stage 1) may achieve wound healing with good wound care and offloading footwear alone; however, the majority of patients with CLTI will require consideration of revascularisation to prevent the need for major lower limb amputation [5]. International guidelines recommend assessment of patient risk, staging of disease using the WIfI score and consideration of anatomical pattern of disease during decision-making in CLTI [5].

Estimation of patient risk in CLTI is challenging, particularly given the poor long-term prognosis of patients with CLTI. The mortality of all patients with CLTI is between 20% and 25% at 1 year, and people with CLTI are now more likely to die than require a major lower limb amputation [5, 8, 46, 47]. Numerous risk calculation tools have been developed, but external validation is poor and uptake in clinical practice is unclear [5]. It is likely that frailty assessment is a useful adjunct to risk estimation given its strong association with mortality in patients with CLTI [8, 28].

### Revascularisation techniques

**Figure 2:**
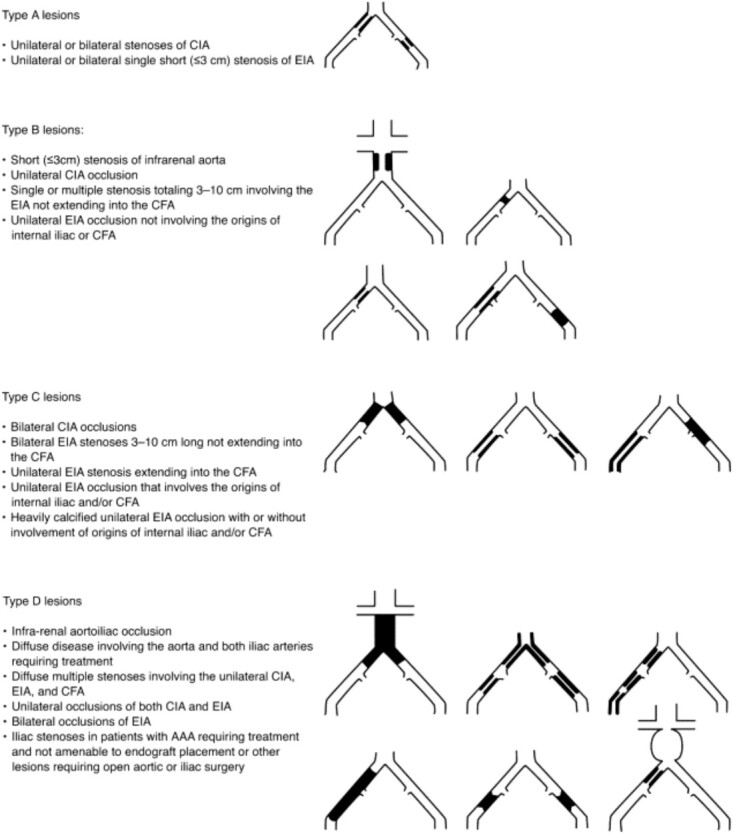
TASC classification for aorto-iliac arterial disease (reproduced with permission from Norgren *et al.* 2007) [48].

**Figure 3:**
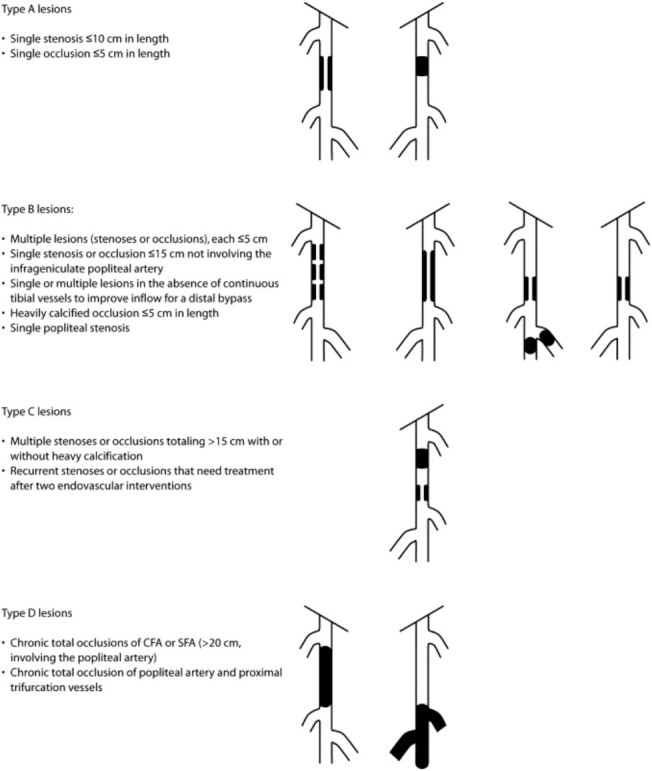
TASC classification for femoro-popliteal arterial disease (reproduced with permission from Norgren *et al.* 2007) [48].

Decisions regarding revascularisation options are also based on anatomical complexity to determine what procedures are technically feasible [5]. The most commonly used anatomical classification system was described in the second Trans-Atlantic Inter-Society Consensus (TASC) for the management of PAD guidelines—with descriptions and classification for both aorto-iliac and femoro-popliteal segment PAD (Figures 2 and 3) [48]. In many cases, there are both endovascular and open surgical options available, and newer technologies are enabling endovascular options for increasingly complex occlusive disease. The advantage of endovascular surgery is that it is minimally invasive and can often be performed under local or regional anaesthetic, reducing perioperative risk and shortening recovery time.

Two recently published randomised trials investigated best treatment options for infra-inguinal disease in CLTI. The BEST-CLI trial demonstrated that open surgical bypass using vein graft is a more durable procedure with fewer re-interventions to maintain patency and a lower major lower limb amputation rate [46]. However, the BASIL-2 trial reported a small but statistically significant worse amputation-free survival at 2 years in patients undergoing lower limb bypass for below-knee arterial disease compared to endovascular revascularisation, due to worse survival in the bypass cohort [47]. A planned individual patient data meta-analysis will aid interpretation of these apparently contradictory results. They do highlight the need for individual assessment of long-term prognosis in shared decision-making as BEST-CLI excluded those with a life-expectancy less than 2 years whilst BASIL-2 only excluded those with a life-expectancy less than 6 months [46, 47].

Newer technologies are pushing the limits of endovascular therapies in CLTI. Covered endovascular revascularisation of the aortic bifurcation (CERAB) is a new technique to recannalise aorto-iliac disease and has promising early results from observational studies [49]. The EVOCC trial recently opened to recruitment and is the first multi-centre randomised trial to compare open and endovascular revascularisation (including CERAB) in patients with CLTI due to aorto-iliac disease. In patients with unreconstructable below-knee arterial disease, endovascular deep venous arterialisation, a new and innovative technique with bespoke technology, has shown early success in preventing major lower limb amputation in observational research [50]. By creating a connection between arteries and veins in the distal leg, oxygenated blood supplies the foot via the venous system with the aim of achieving wound healing. Efficacy of these new technologies is uncertain as is their role in older adults with frailty.

Many patients with CLTI also require wound debridement or minor amputation (toes or forefoot) to aid healing and treat infection. Unfortunately, major lower limb amputation is still often required in patients whom tissue loss is too extensive or in whom revascularisation is not possible or has failed.

### Management pathways in CLTI

The focus of modern management of CLTI is timely revascularisation and, if present, urgent debridement and drainage of severe infection followed by stringent wound management [5]. Delays in the diagnosis, referral and intervention for CLTI may lead to worse outcomes including higher likelihood of major lower limb amputation [51]. Therefore, current best-practice guidelines for management of CLTI in the UK set challenging time targets for revascularisation of 5 days from referral for inpatients and 14 days for outpatients [4]. To help achieve these, many centres in the UK have adopted rapid-access vascular limb salvage clinics to streamline the assessment, investigation and intervention for people referred with suspected CLTI with some evidence of reduction in major lower limb amputation incidence in patients managed in such clinics [3].

### Shared decision-making in CLTI

Shared decision-making can be challenging in CLTI, particularly among older adults given their increased perioperative risk, worse life expectancy and high prevalence of cognitive impairment [8, 9, 27, 28]. Older patients also often have increased care needs after major amputation and are rarely suitable for prosthetic limb fitting [52, 53]. There are competing risks of limb loss (with its subsequent increased care needs and loss of independence) and perioperative morbidity of different revascularisation options; within the context of limited life expectancy. Furthermore, the durability and likelihood of success in relieving symptoms of rest pain, achieving wound healing and improving lower limb function often varies for different revascularisation options [5]. These need to be fully explored with the patient, their relatives and carers during shared decision-making to support their choice of treatment options. Whilst older adults tend to prioritise maintaining independence, remaining in their own home and quality of life over survival, there is very little research on outcomes prioritisation and optimising shared decision-making specifically among those with CLTI [54].

Conservative management of CLTI is often the most appropriate course of action due to a combination of patient (e.g. advanced age or frailty) and anatomical factors (e.g. complex/no revascularisation options) [5]. Optimal medical therapy, wound management and suitable footwear are important components of conservative management of CLTI. Those managed conservatively may have a similar survival to those suitable for revascularisation [55, 56].

Evidence varies regarding the outcomes of revascularisation in older adults. In selected patients undergoing revascularisation, short-term morbidity and mortality are similar in different age groups; however, there is a higher incidence of post-operative delirium in older adults [57, 58]. One-year major amputation incidence is also similar in different age groups in those undergoing revascularisation, but survival is worse among older adults with CLTI [58, 59]. CLTI patients generally demonstrate modest benefits in quality of life after intervention, which is also present in older adults and those with frailty [30, 60, 61].

Patients with extensive infection or gangrene who decline or are not suitable for major amputation may require palliative management. Underutilisation of palliative care has been identified in CLTI patients, and there is a dearth of published evidence for palliative management and discussion of dying in CLTI despite its high mortality [62].

## Perioperative management of patients with CLTI

Given the changing demographics and disease patterns seen in PAD over recent years, patients with PAD admitted and treated on vascular surgery wards (almost exclusively those with CLTI) are increasingly older, frailer and more cognitively impaired with a greater burden of multi-morbidity, polypharmacy and risk of falls and delirium [8–10, 63–65]. A recent survey of Belgian geriatricians ranked vascular surgery as second in a list of surgical specialties most in need of geriatric support, behind only orthopaedics [66]. Expert geriatrician input may be useful to both support shared decision-making, perioperative management and safe discharge in CLTI.

There is emerging evidence of benefit of geriatric liaison services with vascular surgery patients [10, 67–69]. A randomised trial by Partridge *et al.* demonstrated reduction in length of stay and incidence of postoperative delirium in vascular surgery patients who received preoperative comprehensive geriatric assessment (CGA) and optimisation delivered by a perioperative care for older people undergoing surgery (POPS) service [67]. A recent systematic review also showed reduction in incidence of delirium in vascular surgery patients from multi-component preoperative assessment and intervention (e.g. CGA) [69]. Evidence specifically in CLTI is limited though, and delivering solely preoperative interventions is complicated by the need for rapid revascularisation and limited time to institute interventions. An ongoing observational study of prehabilitation in CLTI will give greater insight into its potential benefits and challenges of delivery [70].

Similar improvements in reduction in length of stay and incidence of delirium have also been observed in service level before-and-after observational studies after introduction of geriatric liaison services in vascular surgery patients, over half of whom had PAD [10, 68]. These studies focused on delivery of expert geriatrician review and optimisation of vascular inpatients throughout the perioperative period, similar to the orthogeriatrician model [10, 68]. This model of care is likely to be optimal in CLTI given revascularisation is time-critical and patients often require emergency admission. Updated UK guidance for the provision of vascular surgery services recommends screening for frailty, cognitive impairment and delirium in vascular surgery inpatients [71]. Along with UK best-practice guidance on the management of patient with PAD, these guidelines also recognise the benefit specialist geriatricians (and CGA) can have in supporting both shared decision-making and perioperative care of older or frail patients with CLTI and recommend their inclusion as a core member of the vascular multi-disciplinary team [4, 71]. Further research on implementation of best-practice guidelines is needed to optimise the perioperative management of patients with CLTI that are living with frailty, particularly in the context of limited geriatrician workforce and increasing use of urgent outpatient endovascular revascularisation.

## Conclusions

PAD is common in older people and is a major cause of morbidity and mortality. Increased awareness of PAD and rapid referral of patients with CLTI is likely to improve outcomes and reduce the need for major amputation. Newer technologies are offering minimally invasive options to increasingly complex disease with intervention possible in older, frailer patients. Given the high prevalence of frailty and cognitive impairment in those with CLTI, UK guidelines recommend that older patients undergoing surgery have access to specialist geriatric teams to support their perioperative management.
